# Bridging the Diagnostic Gap in Peripheral Arterial Disease (PAD): Leveraging Fatty Acid Binding Protein 3 (FABP3) for Biomarker-Guided Detection

**DOI:** 10.3390/diagnostics16101457

**Published:** 2026-05-11

**Authors:** Muzammil H. Syed, Abdelrahman Zamzam, Farah Shaikh, Ori D. Rotstein, David J. Klein, Houssam Younes, Rawand Abdin, Mohammad Qadura

**Affiliations:** 1 Division of Vascular Surgery, Department of Surgery, St. Michael’s Hospital, Unity Health Toronto, University of Toronto, Toronto, ON M5B 1W8, Canada; 2 Department of Surgery, University of Toronto, Toronto, ON M5S 1A1, Canada; 3 Institute of Medical Science, University of Toronto, Toronto, ON M5S 1A1, Canada; 4 Li Ka Shing Knowledge Institute, St. Michael’s Hospital, Unity Health Toronto, University of Toronto, Toronto, ON M5B 1W8, Canada; 5 Division of General Surgery, St. Michael’s Hospital, Unity Health Toronto, Toronto, ON M5B 1W8, Canada; 6 Dalla Lana School of Public Health, University of Toronto, 155 College St. Room 500, Toronto, ON M5T 3M7, Canada; 7 Division of General Internal Medicine, Department of Medicine, St. Michael’s Hospital, Unity Health Toronto, University of Toronto, Toronto, ON M5B 1W8, Canada; 8 Heart, Vascular, & Thoracic Institute, Cleveland Clinic Abu Dhabi, Abu Dhabi 112412, United Arab Emirates; 9 Department of Medicine, McMaster University, Hamilton, ON L8S 4L8, Canada

**Keywords:** peripheral arterial disease, diagnostics, fatty acid binding protein 3, biomarker

## Abstract

**Background/Objective**: Peripheral arterial disease (PAD) is a widespread but underdiagnosed manifestation of systemic atherosclerosis, associated with high morbidity and mortality. Traditional diagnostic methods such as the ankle-brachial index (ABI) have limited sensitivity in certain populations, highlighting the need for reliable blood-based biomarkers. Fatty Acid Binding Protein 3 (FABP3) has emerged as a robust biomarker with diagnostic utility in PAD. To evaluate the diagnostic performance of FABP3 when used in combination with traditional clinical risk factors for PAD in patients presenting to vascular surgery clinics. **Methods**: A retrospective analysis was conducted on 657 patients presenting to ambulatory vascular surgery clinics at St. Michael’s Hospital. Two logistic regression models were compared: (1) Model A: Included standard clinical risk factors (calf pain, age, smoking, diabetes, hypertension, hypercholesterolemia, coronary artery disease, and signs of chronic limb-threatening ischemia); and (2) Model B: Included the same factors as Model A, plus FABP3 levels. Diagnostic metrics including area under the curve (AUC), sensitivity, specificity, positive predictive value (PPV), negative predictive value (NPV), and diagnostic accuracy were assessed. **Results**: Among 657 patients, 423 had PAD and 234 did not. Model B (FABP3-integrated model) outperformed Model A, with a higher AUC (0.86 vs. 0.82), sensitivity (96% vs. 81%), specificity (84% vs. 67%), PPV (92% vs. 81%), NPV (94% vs. 65%), and diagnostic accuracy (93% vs. 76%). FABP3 also improved detection in asymptomatic PAD patients (84% detected vs. 0%). **Conclusions**: Integrating FABP3 with standard clinical risk factors significantly improves PAD diagnosis, especially in asymptomatic and borderline cases. These findings support the potential role of FABP3 in routine PAD screening, warranting further prospective studies for validation.

## 1. Introduction

Peripheral artery disease (PAD) is a chronic atherosclerotic disorder, most commonly affecting the arteries of the lower extremities. It is characterized by progressive plaque formation, initially causing arterial narrowing (stenosis) and, in advanced stages, leading to flow-limiting occlusion, which can result in ischemic symptoms such as intermittent claudication or, in severe cases, limb ischemia [1]. It affects an estimated 200 million people globally and is associated with a heightened risk of cardiovascular morbidity, limb loss, and all-cause mortality [1]. PAD often remains undiagnosed until advanced stages due to its insidious onset and the frequent absence of classical symptoms such as intermittent claudication [2,3].

Despite its importance, the current diagnostic landscape for PAD is limited. The ankle-brachial index (ABI), a simple bedside tool comparing systolic pressures in the ankle and arm, is the recommended initial screening test. However, ABI may be falsely elevated in patients with medial arterial calcification, particularly those with diabetes mellitus or chronic kidney disease [4,5]. Additionally, ABI lacks usage in early or asymptomatic disease, leading to missed opportunities for timely intervention. Imaging modalities such as duplex ultrasound, computed tomography angiography (CTA), and magnetic resonance angiography (MRA) offer greater anatomical resolution but are resource-intensive, operator-dependent, and not feasible for large-scale screening or routine follow-up [6,7].

This underscores the unmet clinical need for reliable, minimally invasive biomarkers that can enhance PAD detection and risk stratification. In recent decades, the field of cardiovascular diagnostics has been revolutionized by the introduction of biomarkers such as cardiac troponins, natriuretic peptides, and C-reactive protein (CRP), which have significantly improved the management of myocardial infarction and heart failure [8,9,10]. However, PAD still lacks an analogous biomarker despite its systemic and ischemic nature.

Fatty acid-binding protein 3 (FABP3), also referred to as heart-type fatty acid-binding protein (H-FABP) and mammary-derived growth inhibitor (MDGI), is a low-molecular-weight cytoplasmic protein belonging to the FABP family, which comprises intracellular lipid chaperones involved in the uptake, transport, and intracellular trafficking of long-chain fatty acids [11,12]. FABP3 is predominantly expressed in cardiac myocytes and skeletal muscle cells, reflecting the high oxidative demands of these tissues, where it contributes to the regulation of fatty acid metabolism and cellular energy homeostasis. In addition to its physiological role in lipid handling, FABP3 has been implicated in a range of pathological processes, including myocardial ischemia, heart failure, atherosclerosis, oxidative stress, endoplasmic reticulum stress, and vascular dysfunction [13,14,15,16].

Because FABP3 is abundant in the cytoplasm and rapidly released following cellular injury, circulating levels may rise early after tissue damage, making it an attractive biomarker for acute and chronic cardiovascular conditions [17,18]. This feature has led to substantial interest in FABP3 as a minimally invasive diagnostic and prognostic marker for myocardial injury, and more recently ischemic skeletal muscle injury [15,19,20]. Compared with conventional risk markers, FABP3 may offer additional biological insight into disease activity and tissue-level injury, potentially improving early detection and risk stratification in vascular disease.

In the context of PAD, emerging evidence suggests that FABP3 levels are robustly elevated in patients with the disease and are associated with its severity, highlighting its potential as a valuable tool for risk assessment and disease management [21]. Related work has also suggested diagnostic potential for urinary FABP3, further supporting the relevance of this pathway in PAD biology and clinical assessment [22]. Together, these studies position FABP3 as a biologically plausible and clinically promising candidate biomarker rather than an isolated exploratory observation.

Given the proven utility of FABP3 as a robust diagnostic biomarker for PAD, the clinical integration of FABP3 into PAD diagnostic algorithms remains an area warranting further exploration. This study seeks to address this gap by focusing on assessing the diagnostic performance of FABP3 in conjunction with existing diagnostic modalities for PAD. By investigating how FABP3 complements established diagnostic methods, the aim is to elucidate its role in enhancing the accuracy of PAD diagnosis.

Ultimately, the findings of this study have the potential to inform clinical practice by providing evidence-based insights into the utility of FABP3 in PAD diagnosis. Integration of FABP3 into diagnostic algorithms could offer clinicians a valuable tool for early detection of PAD, enabling early interventions and improved patient outcomes.

## 2. Materials and Methods

### 2.1. Study Participants

This investigation builds upon a previously published, prospectively characterized patient population [21]. In brief, consecutive patients presenting to ambulatory vascular surgery clinics at St. Michael’s Hospital between 4 October 2017 and 30 July 2019 were recruited. This time frame was chosen because it encompassed the period during which comprehensive clinical data, ABI/TBI testing, vascular assessments, and banked plasma FABP3 measurements were available and sufficient to meet statistical power requirements. These patients underwent clinical examination of peripheral pulses and measurement of ankle-brachial index (ABI). PAD was defined as ABI < 0.9 in combination with evidence of atherosclerotic disease on duplex ultrasound, reduced peripheral pulses in at least one leg (with or without claudication). Non-PAD was defined as ABI ≥ 0.9 with palpable distal pulses and no history of claudication. In cases where ABI values were erroneous due to non-compressible tibial vessels (ABI > 1.4), toe brachial index (TBI) measurements were used. Patients with TBI < 0.7 were defined as having PAD.

Patients were excluded if they met any of the following criteria: chronic kidney disease stages 3–5, acute coronary syndrome (ACS), stroke, or transient ischemic attack (TIA) within the past 30 days, or elevated troponin levels, as these conditions could potentially confound plasma levels of FABP3 (Table 1).

### 2.2. Quantification of FABP3 Levels and Analytical Methodology

In concordance with the detailed methodology described in a prior publication [21], sample collection and processing are summarized here. Blood samples were obtained in the morning under fasting conditions; however, non-essential medications were not withheld prior to collection. Samples were drawn into EDTA-containing vacutainer tubes, centrifuged at 3000 rpm for 10 min at 4 °C, and the resulting plasma was aliquoted and stored at −80 °C until analysis. Plasma FABP3 concentrations were measured using the MILLIPLEX MAP Human Cardiovascular Disease (CVD) Magnetic Bead Panel 1 (EMD Millipore, Billerica, MA, USA). To reduce inter-assay variability, plasma samples were analyzed on the same day whenever feasible. The intra-assay coefficient of variability was <10%, whereas the inter-assay coefficient of variability was 15%. Prior to sample acquisition, the MagPix analyzer (Luminex Corp., Austin, TX, USA) was calibrated using Fluidics Verification and Calibration bead kits (Luminex Corp.). At least 50 beads for FABP3 were acquired using Luminex xPONENT software, and analyte concentrations were processed using Milliplex Analyst software version 5.1 (EMD Millipore, Darmstadt, Germany).

### 2.3. Previously Identified Cutoff Points in PAD Diagnosis

Previously reported in a published study [21], cutoff values for FABP3, along with associated sensitivities and specificities, were utilized in this investigation. A cutoff point of <1.55 μg/mL enabled PAD exclusion with 96% sensitivity, yielding a high negative-predictive value (96%) and a negative likelihood ratio of 0.11. Conversely, a confirmatory cutoff point of 3.55 μg/mL provided 95% specificity, yielding an 83% positive-predictive value and a positive likelihood ratio of 10.1 for PAD diagnosis. Patients falling between these cutoffs (1.55–3.55 ng/mL) were in a “grey zone,” necessitating further clinical and radiographical evaluation to ascertain PAD status.

### 2.4. Statistical Analysis

Descriptive statistics were calculated to characterize the baseline demographics and clinical characteristics of the PAD and non-PAD groups. Continuous variables were expressed as means and standard deviations and compared using independent samples *t*-tests or Mann–Whitney U tests where appropriate. Categorical variables were presented as counts and percentages and compared using chi-square tests or Fisher’s exact test.

Two logistic regression models were constructed: Model A included traditional clinical risk factors (calf pain, age, smoking status, diabetes, hypertension, hypercholesterolemia, coronary artery disease, and chronic limb-threatening ischemia (CLTI), as outlined in the 2016 AHA/ACC guideline on Lower Extremity Peripheral Artery Disease management), while Model B included these variables plus FABP3 concentration. These factors were derived from history and physical examination findings suggestive of PAD. Model comparisons were based on metrics such as the area under the curve (AUC), sensitivity, specificity, positive predictive value (PPV), negative predictive value (NPV), and diagnostic accuracy. The Net Reclassification Improvement (NRI) was utilized to assess the enhancement in model performance achieved by adding FABP3.

Subgroup analyses were conducted for symptomatic PAD patients versus non-PAD patients and asymptomatic PAD patients versus non-PAD patients. Statistical significance was determined using a two-sided P threshold of less than 0.05. Data entry and statistical analyses were performed using SPSS software, version 23 (SPSS Inc., Chicago, IL, USA).

## 3. Results

A total of 657 patients were enrolled, of whom 423 were diagnosed with PAD and 234 served as non-PAD controls. The mean age of the total cohort was 66 years (±12), with a male predominance (64%). Comorbidities such as dyslipidemia (69%) and hypertension (67%) were common. PAD patients exhibited a significantly higher prevalence of all cardiovascular risk factors compared to non-PAD patients, with the exception of congestive heart failure, where no significant difference was found. Chronic limb-threatening ischemia was present in 10% of PAD patients. Table 2 summarizes baseline characteristics.

### 3.1. Model Performance: Clinical Risk Factors vs. FABP3-Integrated Model

**Figure 1:**
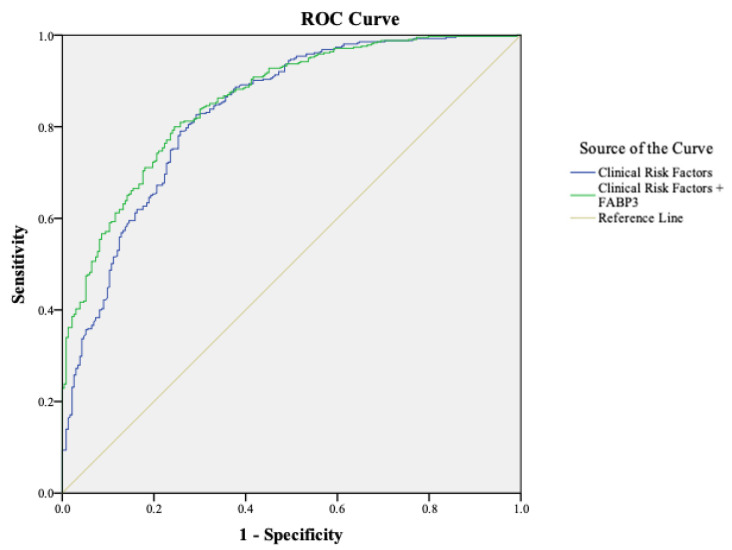
**Comparison of Diagnostic Models.** Comparison of the AUC values between the Model A (Clinical Risk Factors) and Model B (Clinical Risk Factors + FABP3), highlighting the improvement achieved with the addition of FABP3.

Two logistic regression models were evaluated. Model A, which included only clinical risk factors, demonstrated an AUC of 0.82 (95% CI 0.80–0.85). Model B, which integrated FABP3 levels, achieved a significantly higher AUC of 0.86 (95% CI 0.83–0.89). This improvement was supported by a NRI of 0.29 (*p* < 0.001), indicating that FABP3 improved patient classification accuracy beyond traditional clinical features (Figure 1).

Model A yielded a sensitivity of 81%, specificity of 67%, PPV of 81%, NPV of 65%, and overall diagnostic accuracy of 76%. In contrast, Model B outperformed with a sensitivity of 96%, specificity of 84%, PPV of 92%, NPV of 94%, and overall accuracy of 93% (Table 3).

### 3.2. Subgroup Analyses: Asymptomatic vs. Symptomatic PAD

**Figure 2:**
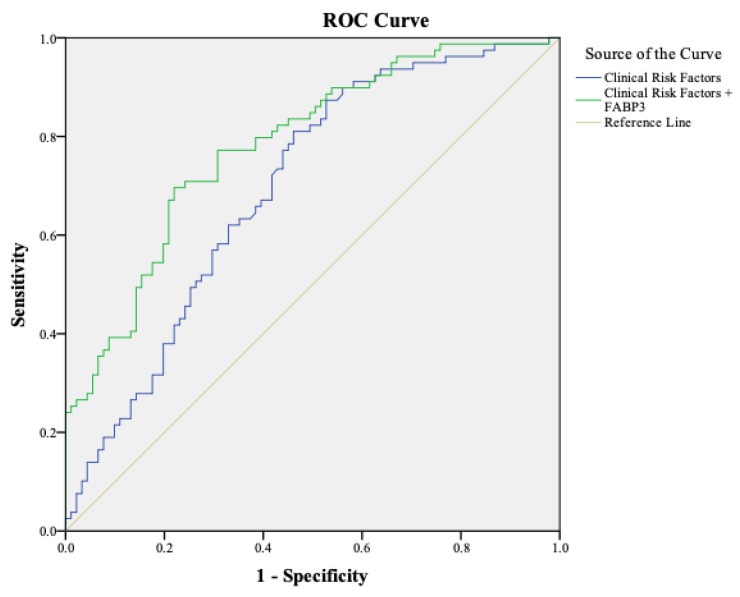
**Diagnostic Performance in Asymptomatic PAD Patients.** Evaluation of the AUC values for the Clinical Risk Factors model and the Clinical Risk Factors + FABP3 model specifically in asymptomatic PAD patients.

In a separate analysis focusing on 173 asymptomatic PAD patients, both logistic regression models were applied for comparison. Model A exhibited an AUC of 0.70 (95% CI 0.66–0.77), while the incorporation of FABP3 (Model B) resulted in an improved AUC of 0.78 (95% CI 0.72–0.85) (Figure 2). The NRI comparing the two models was 0.21 (*p* = 0.019). Notably, Model A failed to detect all 82 asymptomatic PAD cases, while Model B missed only 13, representing an 84% detection rate. Of these 13 missed cases, 54% were moderate PAD and 46% mild, with a mean ABI of 0.71 (±0.10).

**Figure 3:**
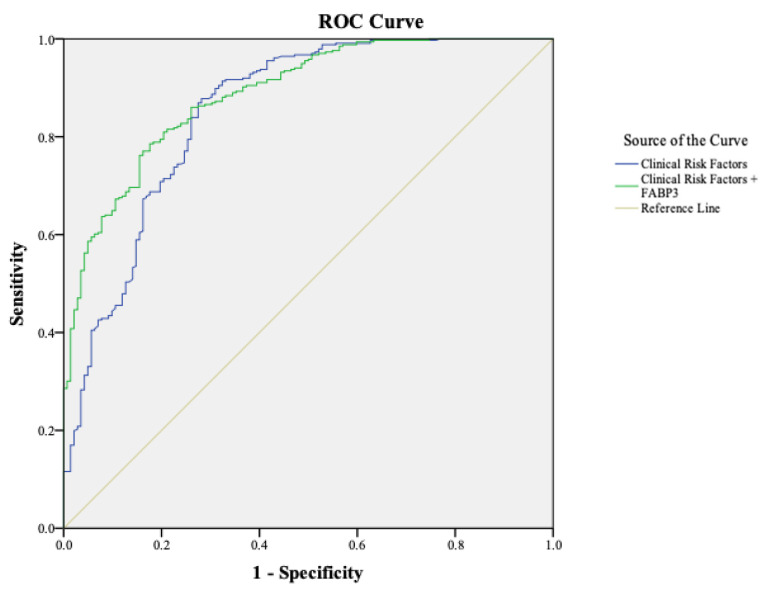
**Diagnostic Performance in Symptomatic PAD Patients.** Comparison of the AUC values for the Clinical Risk Factors model and the Clinical Risk Factors + FABP3 model in symptomatic PAD patients, illustrating the enhanced diagnostic accuracy with FABP3 integration.

Among symptomatic patients (*n* = 484), Model A achieved an AUC of 0.85 (95% CI 0.71–0.89), while Model B improved this to 0.89 (95% CI 0.85–0.92) (Figure 3). The corresponding NRI was 0.17 (*p* = 0.022), indicating added value from FABP3 even in clinically apparent disease.

### 3.3. Grey Zone Analysis (FABP3 1.55–3.55 ng/mL)

**Figure 4:**
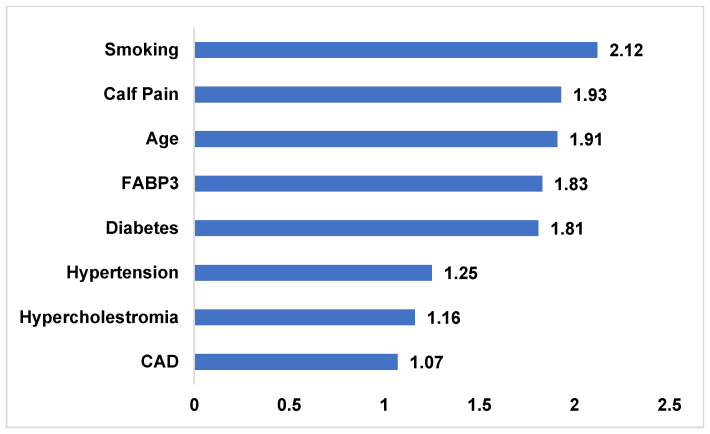
**Determinants of PAD in the Grey Zone.** Identification of smoking, leg pain, and age, as the strongest determinants of PAD within the “grey zone” of diagnosis through logistic regression modeling.

**Figure 5:**
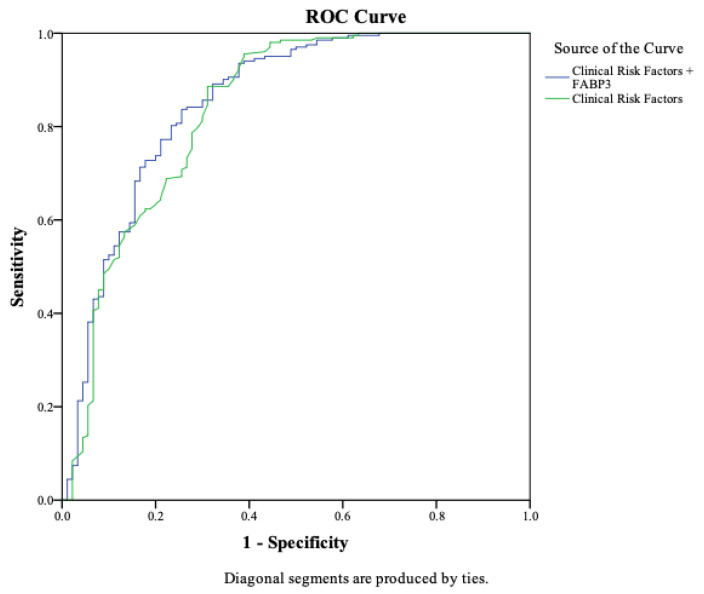
**Enhancement of Diagnostic Accuracy.** Illustration of the improved AUC values with the addition of FABP3 to the Clinical Risk Factors model, emphasizing the enhancement in diagnostic accuracy.

Of the total cohort, 277 patients fell within the grey zone. Among these, 197 had PAD and 80 did not. Regression analysis within this subgroup identified smoking, age, and leg pain as the strongest predictors of PAD (Figure 4). Model A achieved an AUC of 0.83 (95% CI 0.78–0.86), while Model B marginally improved AUC to 0.86 (95% CI 0.82–0.90) (Figure 5). The NRI with Model A as a reference yielded a slight but significant improvement of 0.11 (*p* = 0.031).

## 4. Discussion

The findings of this study underscore the clinical potential of FABP3 as a valuable biomarker in the diagnostic evaluation PAD, particularly when integrated with established risk factors. Incorporation of FABP3 into the diagnostic model resulted in a notable improvement in performance metrics, with the AUC increasing from 0.82 to 0.86. This enhancement was accompanied by substantial gains in sensitivity (81% to 96%), specificity (67% to 84%), PPV (81% to 92%), NPV (65% to 94%), and overall diagnostic accuracy (76% to 93%). Collectively, these findings demonstrate that FABP3 meaningfully strengthens diagnostic precision, supporting its potential utility across both primary care and specialized vascular settings. The integration of cardiovascular biomarkers into clinical practice has revolutionized the diagnosis, screening, and prognostication of various cardiovascular conditions. Well-established biomarkers such as cardiac troponins (cTns), N-terminal pro-B-type natriuretic peptide (NT-proBNP), and C-reactive protein (CRP) play pivotal roles in identifying and stratifying cardiovascular risk [8,9,23]. While cTns are indispensable for detecting myocardial infarction and NT-proBNP for heart failure diagnosis, CRP and other markers serve as valuable prognostic indicators for cardiovascular events. However, despite the strides made in cardiovascular biomarker research, there remains a conspicuous void in specific biomarkers tailored for PAD. This deficiency poses a substantial challenge in the early detection and management of PAD, underscoring the pressing need for further investigations to pinpoint and integrate effective PAD biomarkers into clinical algorithms [24].

### 4.1. FABP3 as a Biomarker for PAD

FABP3 is a 15 kDa cytoplasmic protein abundantly expressed in cardiac and skeletal muscle. It plays a central role in intracellular fatty acid transport and is likely released into the bloodstream following muscle injury or ischemia [25,26]. In the context of PAD, intermittent ischemia due to compromised blood flow in the lower extremities causes repeated bouts of skeletal muscle stress. We hypothesize that as muscle fibers undergo hypoxia-induced damage, FABP3 is released, making it a potential marker of tissue-level ischemia even in the absence of overt clinical symptoms.

Unlike structural imaging modalities, FABP3 reflects dynamic biochemical changes at the cellular level. This positions it as a functional biomarker capable of detecting ischemia-related injury before significant anatomical stenosis becomes radiographically apparent [27]. Similar to how troponins serve as molecular flags of myocardial damage, FABP3 may function as a peripheral “ischemia sensor” for skeletal muscle, signaling underlying PAD well before ABI or imaging are performed.

### 4.2. Clinical Implications

Our results have immediate implications for enhancing PAD screening and diagnosis, particularly in high-risk populations. The improved detection of asymptomatic patients, who often go untreated due to lack of clinical suspicion, suggests that FABP3 can serve as a valuable early screening tool. Asymptomatic PAD has been associated with a significantly increased risk of cardiovascular events, comparable to symptomatic PAD [28]. Early identification and management of this silent subgroup could prevent disease progression, improve quality of life, and reduce downstream complications.

Moreover, FABP3 may be particularly useful in populations where ABI is less reliable. For instance, in patients with diabetes, ABI may yield falsely elevated readings due to arterial stiffening [4]. Our findings indicate that FABP3 can provide supplemental diagnostic clarity in such scenarios. Similarly, smoking, age, and leg pain were found to be strong determinants in the grey zone (FABP3 1.55–3.55 ng/mL), where diagnostic ambiguity is highest. Incorporating FABP3 in these cases improved diagnostic reclassification (NRI = 0.11), underscoring its practical utility.

### 4.3. Limitations

This study represents one of the first attempts to integrate biomarkers into the clinical management of PAD patients, offering novel insights into the potential benefits of a biomarker-driven approach. By complementing traditional clinical risk factors, FABP3 has the potential to enhance diagnostic accuracy, facilitate early detection, and guide personalized treatment strategies in PAD patients. While the findings of this study are promising, this study has several limitations that are important to acknowledge. First, the retrospective design introduces inherent biases related to patient selection and clinical decision-making. Second, this was a single-center study conducted at a tertiary vascular clinic, which may limit the generalizability of findings to broader primary care or community-based populations. Third, FABP3 is not specific to PAD; elevations can occur due to other diseases such as myocardial infarction. Although we excluded patients with recent cardiac events and advanced kidney disease, real-world implementation would require careful interpretation in multimorbid patients.

Another limitation is that we used a single FABP3 measurement per patient. While this approach aligns with most biomarker validation studies, it limits our ability to assess intra-individual variability or temporal trends. Lastly, while FABP3 shows promise as an adjunctive diagnostic biomarker, its efficacy as a standalone indicator and its role in guiding treatment decisions warrant further investigation.

## 5. Conclusions

FABP3 represents a promising and practical biomarker for PAD, capable of enhancing diagnostic accuracy when combined with traditional clinical indicators. It holds particular value for identifying asymptomatic and diagnostically borderline patients, improving early detection and informing timely intervention. While additional prospective validation is essential, our findings mark a critical step toward biomarker-driven precision vascular care.

## Figures and Tables

**Table 1:** Study inclusion and exclusion criteria for PAD and non-PAD participant classification.

Category	Criteria
Inclusion Criteria—PAD group	ABI < 0.9 and evidence of atherosclerotic disease on duplex ultrasound, with reduced peripheral pulses in at least one leg, with or without claudication
Inclusion Criteria—Non-PAD group	ABI ≥ 0.9, palpable distal pulses, and no history of claudication
Special diagnostic criterion for non-compressible vessels	For patients with erroneous ABI due to non-compressible tibial vessels (ABI > 1.4), TBI was used instead
PAD definition using TBI	TBI < 0.7 was considered diagnostic of PAD
Exclusion Criteria	Chronic kidney disease stage 3–5
Exclusion Criteria	Acute coronary syndrome, stroke, or transient ischemic attack within the past 30 days
Exclusion Criteria	Elevated troponin levels

Detailed criteria used to classify participants as PAD or non-PAD. PAD, peripheral arterial disease; ABI, ankle-brachial index; TBI, toe-brachial index.

**Table 2:** Baseline Characteristics of the Study Cohort.

Characteristic	Overall (*n* = 657)	Non-PAD (*n* = 234)	PAD (*n* = 423)	*p*-Value
Age, mean (SD)	66 (12)	60 (13)	69 (10)	<0.001
Male	417 (64)	138 (59)	279 (66)	0.069
Female	239 (36)	96 (41)	143 (34)	
Hypertension	442 (67)	117 (50)	325 (77)	<0.001
Dyslipidemia	456 (69)	114 (49)	342 (81)	<0.001
Diabetes	235 (36)	38 (16)	197 (47)	<0.001
Past smoking	313 (48)	89 (38)	224 (53)	<0.001
Current smoking	168 (26)	39 (17)	129 (31)	<0.001
History of congestive heart failure	15 (2)	5 (2)	10 (2)	0.085
History of coronary artery disease	182 (28)	42 (18)	140 (33)	< 0.001
History of stroke	63 (10)	10 (5)	53 (13)	<0.001
Chronic Limb Threatening Ischemia (CLTI)	43 (7)	0 (0)	43 (10)	<0.001
Leg pain	484 (74)	143 (61)	341 (81)	<0.001

Summary of baseline demographic characteristics of the study cohort, including age, gender distribution, and prevalence of comorbidities.

**Table 3:** Performance Metrics of Diagnostic Models.

*N* = 657	TP	TN	FP	FN	Sensitivity	Specificity	LR+	LR−	PPV	NPV	Diagnostic Accuracy
**ModelA** (Clinical Risk Factors)	341	156	78	82	81%	67%	2.42	0.29	81%	65%	76%
**Model B** (Clinical Risk Factors + FABP3)	410	197	37	13	96%	84%	6.16	0.04	92%	94%	93%

Performance metrics including sensitivity, specificity, positive predictive value (PPV), negative predictive value (NPV), and diagnostic accuracy for both Model A (Clinical Risk Factors) and Model B (Clinical Risk Factors + FABP3).

## Data Availability

The original contributions presented in this study are included in the article. Further inquiries can be directed to the corresponding author.
